# Development and Validation of a High-Performance Liquid Chromatography Method With UV Detection for Determination of 1-(2-phenylethyl)-5- (quinaldin-4-yl) Biuret in Rat Plasma

**Published:** 2013-05-04

**Authors:** Neda Adibpour, Maryam Ahmadnasr, Mohammad Javad Khodayar, Saeed Rezaee

**Affiliations:** 1 Department of Medicinal Chemistry, School of Pharmacy, Ahvaz Jundishapur University of Medical Sciences, Ahvaz, IR Iran; 2 Department of Pharmaceutics, School of Pharmacy, Ahvaz Jundishapur University of Medical Sciences, Ahvaz, IR Iran; 3 Department of Pharmacology and Toxicology, School of Pharmacy, Ahvaz Jundishapur University of Medical Sciences, Ahvaz, IR Iran

**Keywords:** High-pressure Liquid Chromatography, Biuret, chromatography

## Abstract

**Background:**

Recently, biuret derivatives have been reported as showing moderate to good cytotoxic effect against certain cancer cell lines. In this study, a high-performance liquid chromatography method was developed for determination of 1-(2-phenylethyl)-5-(quinaldin-4-yl) biuret (PEQB) in rat plasma to use in future studies on this compound and related derivatives.

**Objectives:**

In this study, we describe a simple and sensitive high-performance liquid chromatography method with UV detection for determination of 1-(2-phenylethyl)-6-(quinaldin-4-yl) biuret (PEQB) in rat plasma.

**Materials and Methods:**

Separations were performed on a Nucleosil-100 CN HPLC column (125 × 4.0 mm) (5 µm), using a mixture of acetonitrile: methanol: potassium dihydrogen phosphate buffer (0.05 M, pH 3.5) (10:10:80) as mobile phase delivered at a flow rate of 1 mL/minute. Detection of PEQB and internal standard (1-([[3-(1,3-benzothiazol-2-ylsulfanyl)propyl]carbamoyl]amino)-N-phenylformamide) was performed at 235 nm and ambient temperature. Plasma samples (200 µL) were prepared by addition of 40 µL internal standard (100 µg/mL), and 400 µL acetonitrile. After vortex mixing and centrifugation at 10000 g, 50 µL of the clear supernatant was directly injected onto the chromatography column. Calibration curves were constructed by fitting the peak area ratio of the biuret to internal standard against concentration of biuret to a power model using generalized least squares nonlinear regression method.

**Results:**

Under the above chromatography condition, biuret compound (PEQB) and the internal standard were detected at 4.5 and 13.5 minutes, respectively. Limit of quantitation of the PEQB was 0.1 µg/mL. Accuracy of the method over the concentration range of 0.1-100 µg/mL was between 88-109%. Inter- and intraday precisions were 4-19% and 6-8%, respectively. A good relationship in the form of a power model was found for two separate concentration ranges of 0.1-1 and 2.5-100 µg/mL (R ^2^> 0.99).

**Conclusions:**

The presented simple HPLC method is sufficiently accurate, precise and sensitive for the quantitation of 1-(2-phenylethyl)-5-(quinaldin-4-yl) biuret in rat plasma.

## 1. Background

**Figure 1.**
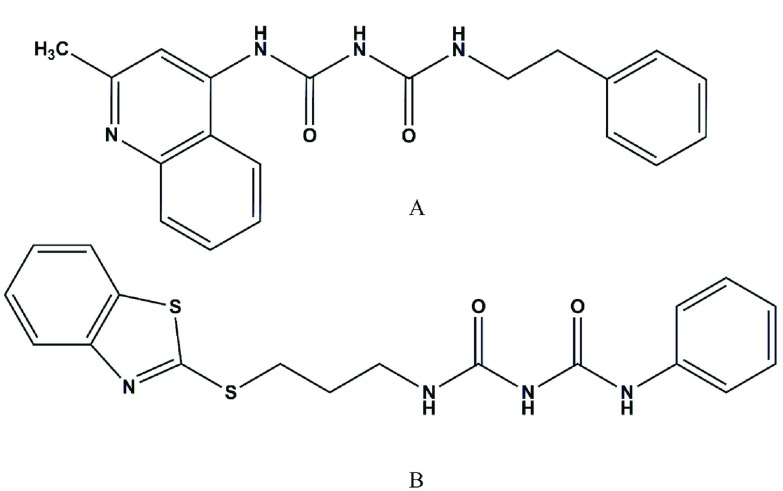
The Structure of 1-(2-phenylethyl)-5-(quinaldin-4-yl) Biuret. B. 1-([[3-(1,3-benzothiazol-2-ylsulfanyl)propyl]carbamoyl]amino)-N-phenylformamide

There are some reports on different pharmacological effects of various derivatives with biuret functionality. For example, synthesis of some phenyl substituted biuret compounds and their effects on gastric acid secretion and inhibition of peptic ulcer has been reported by McColl and his colleagues (1). The hypoglycemic activity of a number of p-toluenesulfonyl-biurets and alkyl p-toluenesulfonyl thiocarbamates has been described by Kriesel et al. (2). In the study of Kajitani and his coworkers, several arylbiurets were prepared and tested as anti-inflammatory and analgesic agents (3). Recently, synthesis and cytotoxicity of N,N`-diphenyl, N-phenyl-N`-alkylphenyl, and N,N`-bisalkylphenyl biurets and analogous compounds by replacing one phenyl group with 2-ethylquinoline-4-yl,benzo[d]thiazol-2-ylthio and (1-phenyl-1H-tetrazol-5-yl) thio moieties against T47D breast cancer cell line have been described. It has been shown that 1-(2-phenylethyl)-5-(quinaldin-4-yl) biuret (Figure 1) is the most cytotoxic biuret derivative on T47D cells (4). An in silico study by Adibpour et al indicated that biuret derivatives could inhibit pteridine reductase 1 of different strains of *leishmania* and could be considered as potential antileishmaniasis agents (5). Since elucidation of the pharmacokinetic profile in early development of a compound is essential and biurets derivatives, as potential chemotherapeutic agents could be the subject of future studies, including assessment of their pharmacokinetics and toxicokinetics in animal models, availability of a simple and sensitive assay method for the analysis of these compounds in biological samples is crucial.

## 2. Objectives

To our knowledge, there is not any reported method for analysis of phenyl biuret derivatives in biological samples or any other matrices, therefore in this study, we describe a simple and sensitive high-performance liquid chromatography method with UV detection for determination of 1-(2-phenylethyl)-6-(quinaldin-4-yl) biuret (PEQB) in rat plasma.

## 3. Materials and Methods

### 3.1. Chemicals and Reagents

1-(2-Phenylethyl)-5-(quinaldin-4-yl biuret and 1-([[3-(1,3-benzothiazol-2-ylsulfanyl)propyl]carbamoyl]amino)-N-phenylformamide used as internal standards were synthesized as previously reported (4), and further purified by preparative thick layer chromatography using silica gel (60 HF254-366) as stationary phase and a mixture of chloroform-ethyl acetate (3:1) as a mobile phase, and recrystallization. For recrystallization, the sample was first dissolved in a minimum amount of warm ethyl acetate, and then recrystallized with cooling the mixture and adding n-hexane portion by portion up to complete precipitation of the biuret compounds .Methanol and acetonitrile were of chromatography grade and purchased from Merck (Germany).

### 3.2. Preparation of Standards and Quality Control (QC) Samples

The stock standard solutions of PEQB biuret and the internal standard were prepared separately by dissolving the accurately weighted standard compounds in methanol to give final concentrations of 1000 µg/mL of both compounds and stored at -20 °C. The stock solutions were further diluted with methanol to achieve the spiking working standards. The spiking standard solutions (20 μL) were used to spike blank rat plasma samples (180 μL), for both calibration curves and QC samples during method validation study. All the working standards were kept at 4 °C until the time of analysis.

### 3.3. Chromatography Conditions

The chromatography system consisted of a Waters ® binary HPLC pump 1525, Waters ® 2487 dual wave length UV-visible detector and Breeze ® software for data acquisition and integration (Waters ® Corporation, USA). Chromatography was performed on a Nucleosil-100 ® CN column (125 mm × 4.0 mm i.d., 5 μm particle size) fitted with an integrated guard column. PEQB and the internal standard were detected at 235 nm. The optimized mobile phase was a mixture of acetonitrile: methanol: potassium di-hydrogen phosphate buffer (0.05 M, pH 3.5) (10:10:80) delivered at the flow rate of 1 mL/minute. Chromatography was performed at ambient temperature.

### 3.4. Sample Preparation

Plasma samples (200 µL) were treated by addition of 40 µL internal standard (100 µg/mL) and 400 µL acetonitrile. After vortex mixing and centrifugation at 10000 g, 50 µL of the clear supernatant was directly injected onto the chromatography column.

### 3.5. Method Validation

Analytical method validation was conducted according to the FDA guidance for bio analytical method validation (6). Selectivity of the method was assessed by comparing different chromatograms of rat plasma blank samples with those plasma samples spiked with PEQB and the internal standard and confirming that there were no interference peaks at the retention times of both external and internal standards. Calibration curves were constructed by plotting the peak area ratio of the PEQB to the internal standard against concentration of PEQB in plasma standard samples. Calibration curves were fitted using piecewise regression followed by generalized least squares regression method. Standard curves were assessed over the concentration range of 0.1-100 µg/mL of the tested biuret. The analytic concentrations corresponding to signal to ratio of 3 and 10 were considered as limits of detection and quantitation, respectively. To confirm the linearity of the calibration curves, the accuracy of back calculated quality control plasma samples should be within ± 15% (± 20% for limit of quantitation) at least for 75% of the nonzero control samples. Interday accuracy and precision were determined by analyzing four QC samples on five different days. Intraday accuracy and precision were evaluated by analyzing five replicates at four different concentrations of QC samples. Accuracy, expressed as percent of calculated concentration to the nominal concentration of the QC samples should be within 75-115% (8-120% for limit of quantitation). Precision values were expressed as coefficients of variations and should be less than 15 % (or 20% for limit of quantitation). Determination of recovery was performed by comparing the peak areas of the extracted QC plasma samples (with the above explained clean up method) at four levels of biuret standards to the unextracted standards ( nonplasma working standards prepared by mixing corresponding methanolic working standard with the same amount of acetonitrile ad internal standard used in cleaning up the plasma samples). Storage stability evaluation was performed by analyzing four standard samples stored at relevant storage conditions. The results should be within 15% of the nominal concentrations. The stabilities of standard samples were also assessed after three cycles of freezing (at -20 °C in 24 hours) and thawing at room temperature.

### 3.6. Administration of PEQB to Rats and Blood Sampling

To assess the ability of the proposed method to quantify PEQB in rat plasma for pharmacokinetic study purposes, the tested biuret was given to three Wistar rats (weight between 200 and 250 g) at the dose of 4 mg/kg via different routes of administration (oral, intravenous and intraperitoneal). Blood samples were collected in citrate tubes from a tail vein at different times up to eight hours post administration.

## 4. Results

UV spectrum of PEQB shows to maxima at 235 and 296 nm. To increase the sensitivity of the method UV detection was performed at 235 nm. Using C18 and C8 stationary phase resulted in either long retention times or broad and bad-shaped peaks. Cyanopropyl column with the aforementioned mobile phase led to well-resolved peaks with reasonable retention times.

**Figure 2.**
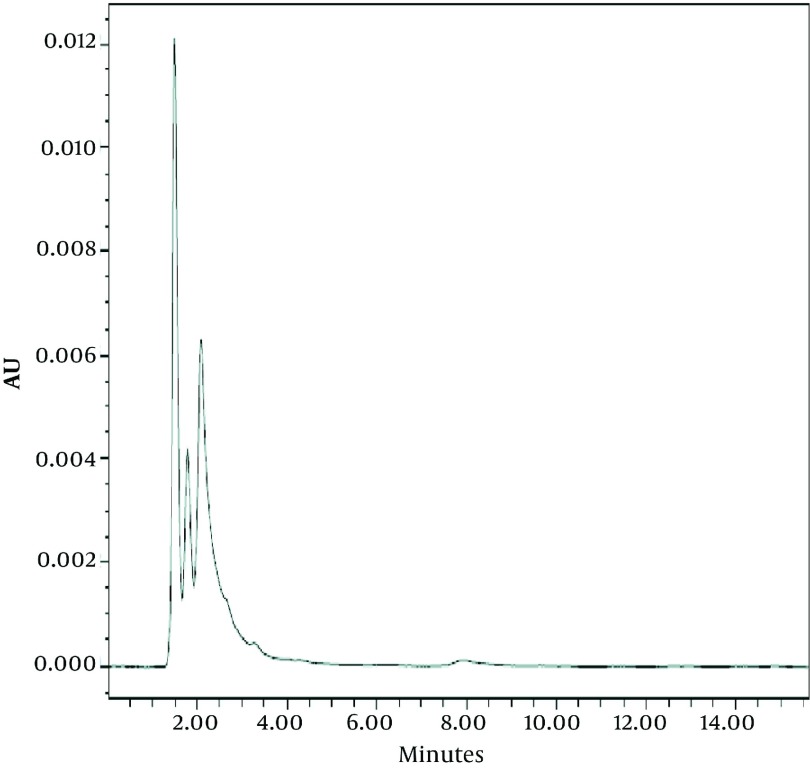
Chromatogram of the Rat Blank Plasma Sample

**Figure 3.**
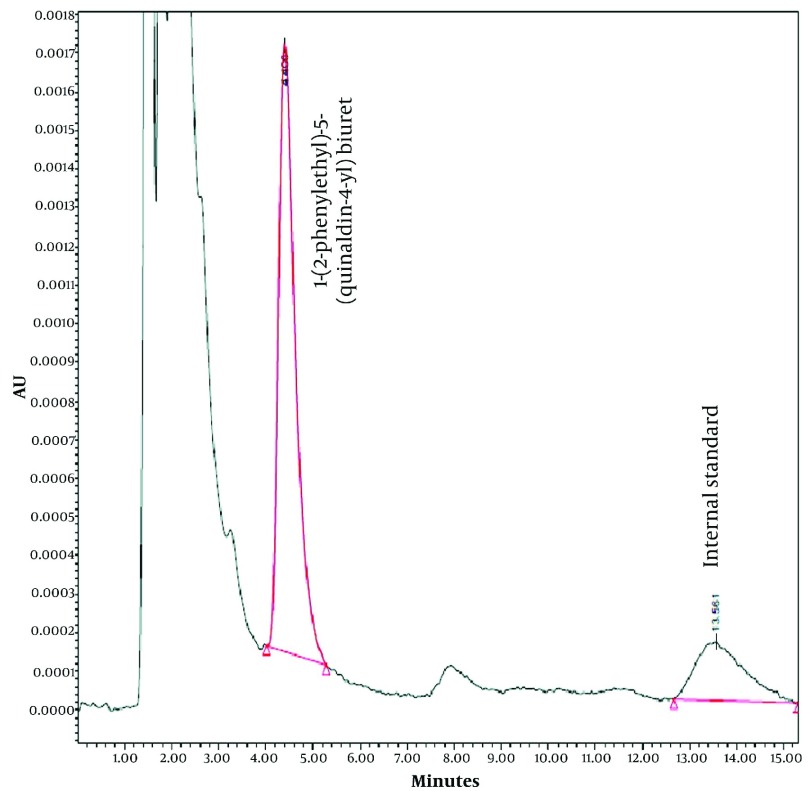
Chromatogram of Plasma Standard Sample at Concentration of 5 µg/mL

The selected internal standard (1-([[3-(1,3-benzothiazol-2-ylsulfanyl) propyl] carbamoyl]amino) -N-phenylformamide is another biuret derivative (4) which is well separated from endogenous compound and PEQB. As could be seen from the chromatogram of rat blank plasma (Figure 2) there was no remarkable interference of endogenous compounds with PEQB and the internal standard. Under the optimized chromatography condition, the retention times of the PEQB and the internal standard were about 4.5 and 13.5 minutes, respectively (Figure 3).

Results of different methods of extraction such as liquid-liquid extraction with ethyl acetate, dichloromethane, diethyl ether, and direct protein precipitation with methanol led to either low recovery or peak broadening. Direct precipitation of plasma proteins with acetonitrile resulted in good recovery and peak shape. Piecewise regression analysis of the calibration standards revealed that two different power models in the form of PAR = a.Cb could be fitted to peak area ratio against concentration of the biuret compound. In the above equation PAR is the peak area ratio of the external to internal standard, C is the concentration of the standard samples of PEQB, and “a” and “b” are the parameters of the model equation (Table 1).

**Table 1.** Parameters of the Standard Curve Equations (PAR = a.Cb)

Parameter	PAR a < 1.1			PAR > 1.1		
	Value	SE b	P-value	Value	SE	P-value
**a**	0.1619	0.0196	< 0.0143	0.5061	0.0201	< 0.0001
**b**	1.0106	0.0848	< 0.0070	0.9512	0.0077	< 0.0001
**Weight Factor c**	1/C ^2.081^			C ^3.148^		
**R ^2^**	0.993			0.994		

^a^Peak Area Ratio of 1-(2-phenylethyl)-5-(quinaldin-4-yl) Biuret to Internal Standard

^b^Standard Error of the Parameter

^c^Wight Factor Estimated by Generalized Least Squares Regression Method

**Figure 4.**
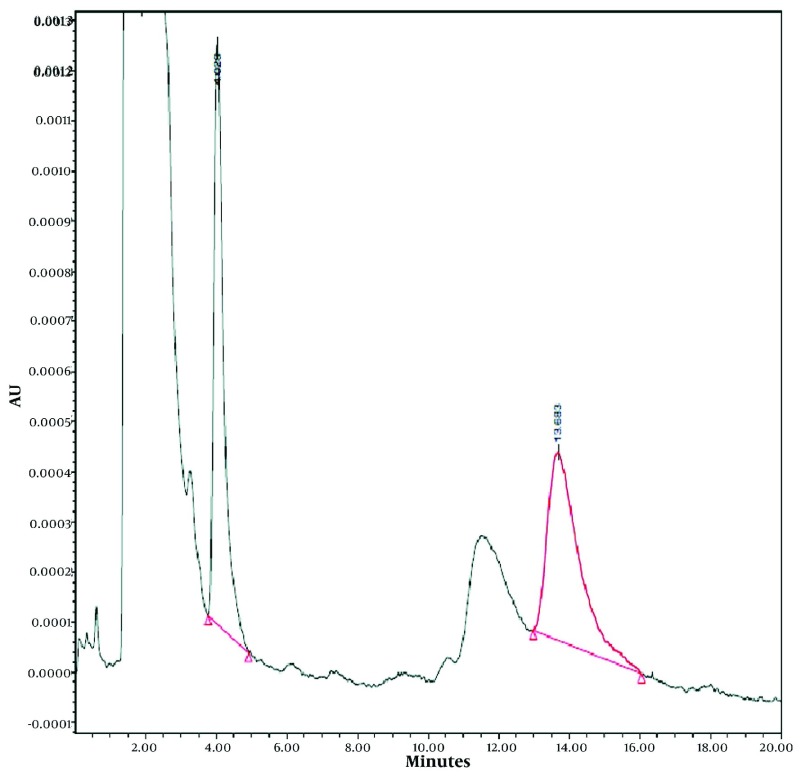
Chromatogram of Plasma Sample Obtained Two HoursAfter the Oral Administration of 4 mg/kg of 1-(2-phenylethyl)-5-(quinaldin-4-yl) Biuret to a Rat (Equal to 1.1 µg/mL)

**Figure 5.**
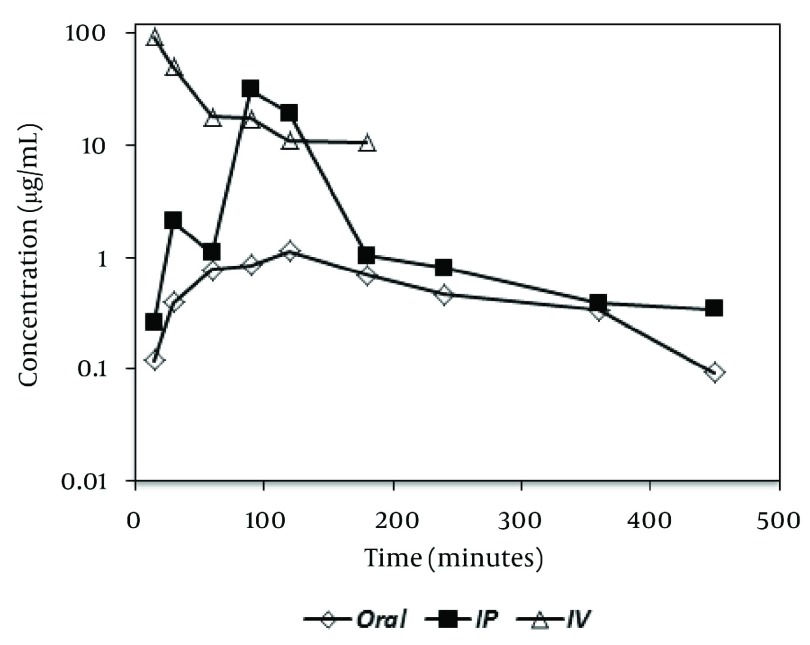
Plasma Concentration-time Profiles of 1-(2-phenylethyl)-5-(quinaldin-4-yl) Biuret After Administration of 4 mg/kg to Rat Via Oral, Intraperitoneal (IP), and Intravenous (IV) Routes of Administration to Three Different Rats

The limits of detection and quantitation of the presented HPLC method were 0.03 and 0.1 µg/mL, respectively. As could be seen from the results presented in Table 2, the accuracy of the method was between 88% and 109%. Inter and intraday precision are smaller than 13% for concentrations other than the limit of quantitation. Recovery values for four levels of PEQB and the internal standard at the concentration resulted from the addition of 40 µL of its 100 µg/mL standard solution to 200 µL plasma are also presented in Table 2. Results of analysis of all QC samples checked during the stability studies at different conditions (including three cycles of freeze and thaw) were within 15% of the initial nominal concentration, and no sign of significant instability was detected. Figure 4 shows chromatogram of a plasma samples obtained two hours after the oral administration of biuret compound to a rat. Plasma concentration-time profiles of the administered biuret derivative via different routes were depicted in Figure 5.

**Table 2.** Recovery, Intra- and Interday Accuracy and Precision Results (n = 5)

Nominal Concentration, µg/mL	Precision, %		Accuracy, % ± SD		Recovery, % ± SD
	Intra - day	Inter - day	Intra - day	Inter - day	
**0.1, LOQ**	8	19	91 ± 7	88 ± 13	73 ± 11
**1**	6	13	103 ± 5	107 ± 9	84 ± 5
**2.5**	8	4	105 ± 8	109 ± 4	83 ± 12
**100**	6	3	96 ± 9	100 ± 12	89 ± 6
**Internal Standard, 6.25 µg/mL a**	-	-	-	-	95 ± 11

^a^Final Concentration of Internal Standard After Mixing 40 µL (100 µg/mL) With 200 µL Plasma Samples and 400 µL Acetonitrile

## 5. Discussion

There is no report on HPLC analysis of similar compounds with biuret functionality and comparable chromophore groups. UV detection was used based on the presence of several aromatic rings in the structure of the tested compound. A number of HPLC column including C18, C8, and CN were tested as stationary phases. High lipophilicity of the PEQB led to either long retention times or broad and bad-shaped peaks on C18 and C8 columns. Use of cyanopropyl column and the optimized mobile phase resulted in elution of both the PEQB and the internal standard at reasonable retention times with good peak shape. Different extraction procedures such as liquid-liquid extraction with ethyl acetate, dichloromethane, diethyl ether and direct protein precipitation with either methanol or acetonitrile were assessed. Among these methods of sample clean up, protein precipitation with acetonitrile was selected because of its simplicity, good peak shapes, and acceptable recovery of the analytes. It has been reported that acetonitrile in the ratio of 2:1 of acetonitrile to plasma sample could result in protein precipitation up to 96 percent of total plasma proteins (7). Several phenyl biuret derivatives were tested to be used as internal standard among which (1-([[3-(1,3-benzothiazol-2-ylsulfanyl)propyl]carbamoyl[amino)-N-phenylformamide showed good separation from PEQB, and was well resolved from the endogenous peaks. Multiphasic disposition kinetics was detected following the administration of PEQB to rats which could be related to lipophilic nature of the biuret derivative. Also, double peak was seen after oral dose of the biuret compounds.

However, further investigation is needed to elucidate the exact pharmacokinetic behavior of this phenyl biuret compound. It is concluded that the presented simple HPLC method is sufficiently accurate, precise, and sensitive for the quantitation of 1-(2-phenylethyl)-5-(quinaldin-4-yl) biuret in rat plasma. It could be also used for determination of other phenyl biuret derivatives with some modifications.
